# An engineered miniACE2 protein secreted by mesenchymal stromal cells effectively neutralizes multiple SARS-CoV- 2 variants in vitro

**DOI:** 10.1186/s10020-025-01190-w

**Published:** 2025-04-23

**Authors:** Sara Moreno-Jiménez, Gina Lopez-Cantillo, Jenny Andrea Arevalo-Romero, Ana María Perdomo-Arciniegas, Andrea Marisol Moreno-Gonzalez, Bellaneth Devia-Mejia, Bernardo Armando Camacho, Paulino Gómez-Puertas, Cesar A. Ramirez-Segura

**Affiliations:** 1 Unidad de Ingeniería Celular y Molecular, Instituto Distrital de Ciencia, Biotecnología e Innovación en Salud, IDCBIS, 111611 Bogotá, Colombia; 2 Research and Innovation Area, Laboratorio Nacional de Diagnostico Veterinario, Instituto Colombiano Agropecuario, 110221 Bogotá, Colombia; 3 Banco de Sangre de Cordón Umbilical, BSCU, Instituto Distrital de Ciencia, Biotecnología e Innovación en Salud, IDCBIS, 111611 Bogotá, DC Colombia; 4 Grupo de Modelado Molecular del Centro de Biología Molecular Severo Ochoa, CSIC-UAM, 28049 Madrid, Spain

## Abstract

SARS-CoV- 2 continues to evolve, producing novel Omicron subvariants through recombinant lineages that acquire new mutations, undermining existing antiviral strategies. The viral fitness and adaptive potential of SARS-CoV- 2 present significant challenges to emergency treatments, particularly monoclonal antibodies, which demonstrate reduced efficacy with the emergence of each new variant. Consequently, immunocompromised individuals, who are more susceptible to severe manifestations of COVID- 19 and face heightened risks of critical complications and mortality, remain vulnerable in the absence of effective emergency treatments. To develop translational approaches that can benefit this at-risk population and establish broader therapeutic strategies applicable across variants, we previously designed and engineered in silico miniACE2 decoys (designated BP2, BP9, and BP11). These decoys demonstrated promising efficacy in neutralizing Omicron subvariants. In this study, we leveraged the therapeutic potential of mesenchymal stromal cells (MSCs) for tissue repair and immunomodulation in lung injuries and used these cells as a platform for the secretion of BP2. Our innovative assays, which were conducted with the BP2 protein secreted into the culture supernatant of BP2-MSCs, demonstrated the potential for neutralizing SARS-CoV- 2, including Omicron subvariants. The development of these advanced therapeutic platforms holds significant promise for scalability to effectively mitigate the impact of severe COVID- 19, contributing to broader and more resilient treatment strategies against the evolving landscape of SARS-CoV- 2 variants.

**Supplementary Information:**

The online version contains supplementary material available at 10.1186/s10020-025-01190-w.

## Background

SARS-CoV- 2 has marked the most significant pandemic event of the twenty-first century. Although the official emergency phase of the pandemic has ended, the virus continues to evolve, acquiring mutations that enhance its spread and persistence. One of the most recent Omicron subvariant characterized, XEC, has multiple mutations in the Spike protein and in its N-terminal domain, with a breakpoint at genomic position 21,738–22,599. These genetic events are linked to increased transmissibility and infectivity, as well as a greater ability to evade immune responses than their predecessors (Kaku et al. 2024; Medicine 2024; Liu et al. 2025).

In patients with COVID- 19, the lungs are among the first and most severely affected organs. This is primarily due to the binding of the virus to the angiotensin-converting enzyme 2 (ACE2) receptors found on alveoli, where cytokine storms can induce damage (Su et al. 2021; Shaikh et al. 2024). Owing to the effects of SARS-CoV- 2 on the respiratory tract, the use of mesenchymal stromal cells (MSCs) is a promising therapeutic strategy for lung tissue repair (Arevalo-Romero et al. 2024). MSCs can regulate the inflammatory environment, promote endogenous repair and reduce the occurrence of a “cytokine storm”, which are the major causes of organ damage that leads to the progression of severe COVID- 19 (Shi et al. 2021; Li et al. 2024a). The safety and efficacy of MSC therapies have been demonstrated in many clinical trials conducted to evaluate the immunomodulatory capacity of MSCs to prevent or attenuate pneumonia and the cytokine storm triggered by COVID- 19 (Shi et al. 2021; Leng et al. 2020; Shetty 2020; Atluri et al. 2020; Liang et al. 2020; Metcalfe 2020; Hou et al. 2024; Li et al. 2024b). These multipotent cells have a high proliferation rate and can be derived from various sources (e.g., bone marrow and the umbilical cord). One of the advantages of MSCs is that they are easily expanded to clinical volumes. MSCs can be stored for repeated use, show no adverse reactions in clinical trials, and have documented safety and efficacy profiles (Golchin et al. 2020; Marquez-Curtis et al. 2015).

To combat severe COVID- 19, a promising strategy involves leveraging the immunomodulatory and anti-inflammatory properties of MSCs (Saeedi et al. 2019; Pittenger et al. 2019) alongside neutralizing peptides or monoclonal antibodies (mAbs). This combined approach could lead to a cellular therapy that limits viral infection in ACE2 + cells, reducing severe lung inflammation, which is associated with the promotion of tissue regeneration in affected patients (Wang et al. 2023). Previously, we engineered miniACE2 peptides (BP2, BP9, and BP11) that demonstrated neutralizing activity against SARS-CoV- 2 variants (Arévalo-Romero et al. 2024). In this work, MSCs were modified to secrete BP2, which targets the spike protein RBD to block viral entry. This approach holds potential as a feasible and broader cellular therapy platform to counteract SARS-CoV- 2 emerging variants, especially in high-risk populations.

## Methods

### Isolation of MSCs from Wharton's jelly

Umbilical cord fragments, approximately 10 cm in length, were collected from three donors. This procedure was approved by the Ethics Committee of *Subred Integrada de Servicios de Salud Norte de Bogotá* (Acta 47 CEI), and informed consent was obtained from each patient. The tissues were preserved in sterile phosphate-buffered saline (PBS) containing 1% penicillin‒streptomycin (P/S) (Gibco^™^, Thermo Fisher, Waltham, MA, USA) and stored at 4 °C until processing. The isolation process started within a maximum of 12 h post-collection. Cell isolation was based on the spontaneous migration of fibroblast-like cells from Wharton's jelly explants cut into 2–5 mm fragments. The cell culture was maintained at 37 °C in a humidified atmosphere with 5% CO_2_ and atmospheric oxygen, after which the cells were cultured in Dulbecco’s modified Eagle’s medium (DMEM) (Gibco^®^, Thermo Fisher, Waltham, MA, USA) supplemented with 10% fetal bovine serum (FBS) without antibiotics. The medium was replaced every 72 h until fibroblast-like cells adhered, and colonies formed. These cells were then expanded and cryopreserved.

### Immunophenotypic characterization of MSCs by flow cytometry

The immunophenotypic characterization of MSCs was performed via a flow cytometer (BD FACSCanto II, BD Biosciences, San Jose, CA, USA) following the guidelines of the International Society for Cell Therapy (ISCT). Markers with high expression in MSCs, including CD90-fluorescein isothiocyanate (FITC-exvio), CD105-allophycocyanin (APC-BioLegend), and CD73-allophycocyanin (APC-exvio), were used for positive identification. Additionally, the following markers with low or no expression in MSCs were analyzed: HLA I-fluorescein isothiocyanate (FITC-BioLegend), CD45-V500 (Becton Dickinson), CD34-allophycocyanin (APC-exvio), CD31-allophycocyanin (APC-BioLegend), CD14-V450, and HLA-DR-V450 (Becton Dickinson).

### Design of the BP2-expressing lentiviral plasmid

A third-generation lentiviral vector backbone was chosen for its enhanced safety profile. A BP2-expressing lentiviral plasmid was constructed to facilitate the efficient production and delivery of the BP2 protein. The BP2 gene sequence, optimized for human expression, was placed under the control of the human eukaryotic translation elongation factor 1 alpha (hEF- 1α) promoter to drive its expression. To increase translational efficiency, a Kozak sequence was added upstream of the start codon.

**Fig. 1:**
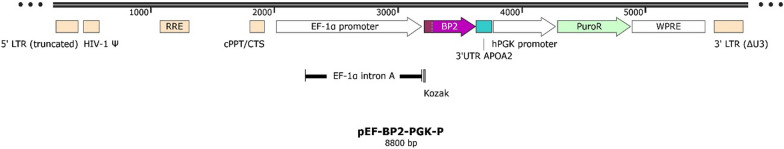
Schematic representation of the third-generation lentiviral vector pEF-BP2-PGK-P, designed for BP2 miniACE2 protein secretion in MSC supernatants. The BP2 gene, which was optimized for human expression, was driven by the hEF- 1α promoter with a Kozak sequence for efficient translation. An IL- 2 signal peptide enables protein secretion, whereas the APOA2 3′ UTR enhances mRNA stability. Puromycin resistance, which is controlled by the hPGK promoter, facilitates selection. The plasmid was synthesized by Gene Universal (Newark, DE, USA)

To facilitate BP2 protein secretion into the supernatant, the IL- 2 signal peptide was included at the N-terminus of the BP2 sequence. Additionally, the *Homo sapiens* apolipoprotein A2 (APOA2) 3′ UTR was introduced downstream of the BP2 stop codon to increase mRNA stability. For the efficient selection of transduced cells, a puromycin resistance gene was driven by the human phosphoglycerate kinase (hPGK) promoter. The complete lentiviral plasmid was synthesized by Gene Universal (Newark, DE, USA) and is detailed in Fig. 1.

### Production of lentiviral vectors

Expi293 cells, adapted for suspension culture, were grown in CTS™ LV-MAX™ Production Medium (Gibco, Cat. #A4124001). The cells were directly thawed into this medium and cultured at 37 °C with ≥ 80% relative humidity and 8% CO₂ on an orbital shaker at 110 rpm. The cells were maintained until they reached a viability of ≥ 95% and a cell density of 3.5–5.5 × 10⁶ viable cells/mL.

For transfection, LV-MAX^™^ Lentiviral Packaging Mix (LPM: Gibco, Cat. #A43237) was used with a plasmid DNA concentration of 2.5 μg/mL relative to the culture volume. A 3:2 ratio of LPM (Invitrogen CAT # A43237) to the Lentiviral Transfer Vector (LTV) was maintained, corresponding to 1.5 μg of LPM per 1 μg of LTV. The cells were transfected at a density of 4.0 × 10⁶ viable cells/mL under the same culture conditions. Six hours post-transfection, the CTS^™^ LV-MAX^™^ Enhancer (Gibco, CAT#A35348) was added to the culture.

The lentiviral vector-containing supernatant was harvested 52 h post-transfection. The culture medium was collected and centrifuged at 600 rpm for 10 min, and the supernatant was filtered through a 0.45 µm PVDF (Millex-HV Cat: SLHU033RS) filter. A Lenti-X™ Concentrator (TAKARA CAT# 631232) was used to concentrate the virus according to the manufacturer's instructions. Briefly, 1 volume of Lenti-X^™^ Concentrator was added to 3 volumes of the supernatant. The mixture was incubated at 4 °C for 4 h, followed by centrifugation at 1500 × g for 45 min at 4 °C. The resulting pellet was resuspended in 1/100 of the original volume in LV-Max production medium and stored at − 80 °C for the subsequent transduction of MSCs.

### Cell culture and engineering

MSCs obtained from Wharton’s jelly were maintained at 37 °C in DMEM supplemented with 5% FBS, 0.5 mM sodium pyruvate (Gibco CAT# 11360070), 7.5 µg/ml nicotinic acid (Sigma Aldrich, CAT#. 8187140100), 50 µg/ml β-nicotinamide adenine dinucleotide hydrate, free acid (β-NAD: Roche, Cat. 10127990001), 50 µM β-mercaptoethanol (βME: Sigma Aldrich, CAT# M6250) and 0.5% HyClone^™^ Cell Boost^™^ 5 Supplement (Cytiva CAT# SH30865.01). The MSCs were incubated under these conditions for 4 days.

**Fig. 2:**
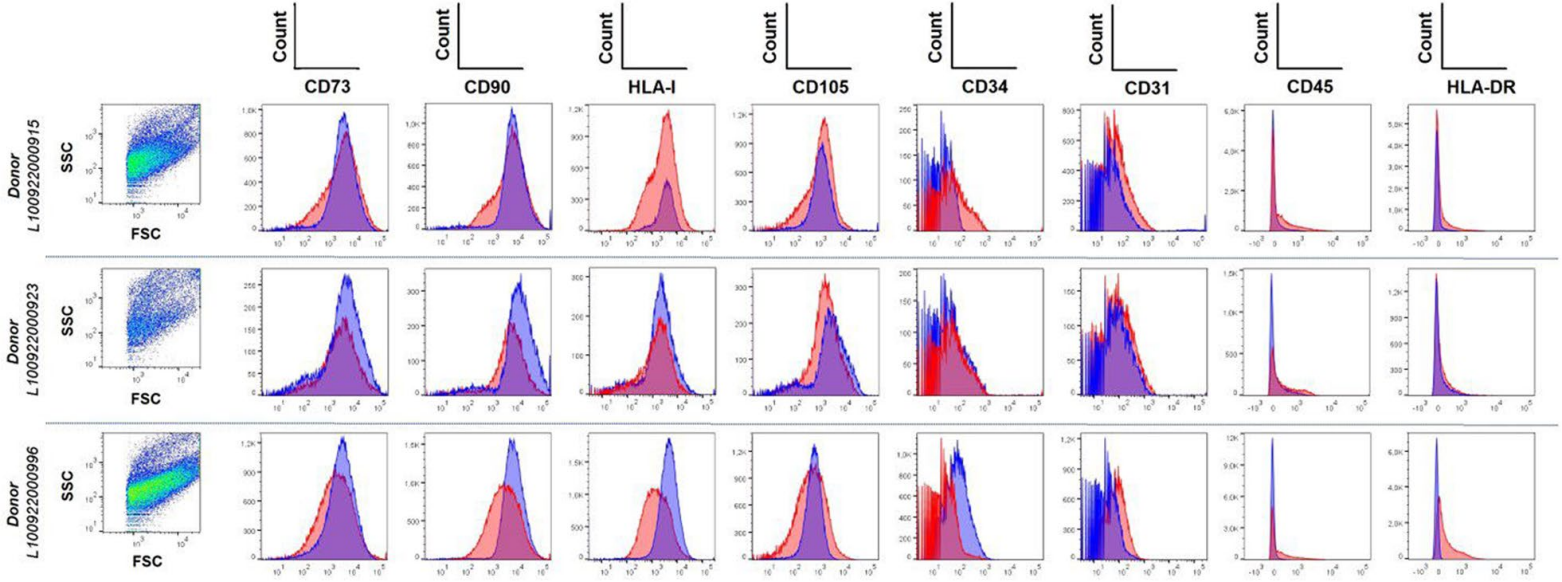
Immunophenotyping of wild-type and BP2-transduced MSCs. Forward and side scatter density plots are shown for wild-type MSCs from each donor. Wild-type MSCs (blue) and BP2-transduced MSCs (red). Data were acquired via a FACSCanto II flow cytometer (Becton Dickinson) and analyzed with FlowJo V10 (TreeStar Inc., USA)

MSCs were transduced with the pEF-BP2-PGK-P lentiviral vector in the presence of 50 µg/ml protamine sulfate (II, Sigma‒Aldrich CAT #P4380 - 100G) to increase the transfection efficiency, resulting in three BP2-secreting MSC strains (BP2-MSC0996; BP2-MSC0923; and BP2-MSC0915) (Fig. 2). After transduction, the MSCs were given a 3-day recovery period in medium supplemented with 10% human platelet lysate (hPL). To select transduced cells, MSCs were cultured under selective pressure with increasing puromycin concentrations (0, 0.5, 1 and 2 µg/ml). The cells with the highest resistance (2 µg/ml) at passage 7 were frozen in liquid nitrogen for subsequent experiments.

### ELISA neutralization assay

To reduce the risk of potential protein degradation, we used the cell supernatant directly from the CPass^™^ SARS-CoV- 2 Neutralization Antibody Detection Kit without prior concentration (Nanjing GenScript Diagnostics Technology Co., Jiangsu Province, China, Inc.). Despite this approach, BP2 protein levels in the supernatant were barely detectable by Western blot analysis. To increase the sensitivity of the cPass assay, we tested two different RBD-HRP/supernatant ratios, 4:2 and 4:3, as detailed below. The neutralization threshold of the assay was set at 30%, as defined by the manufacturer's specifications. Values equal to or above 30% were considered indicative of neutralization, whereas values below 30% indicated the absence of neutralization.

Supernatants from wild-type MSCs (wtMSCs) and BP2-MSCs derived from three different donors were evaluated in this study. All the experiments were performed according to the manufacturer’s instructions. MSC supernatants were first prefiltered via a 0.45 µm filter and subsequently diluted in the provided dilution buffer. Two distinct dilutions of the supernatants were prepared: 30 µL of the supernatant was mixed with 30 µL of dilution buffer, and 45 µL of the supernatant was mixed with 15 µL of dilution buffer. Next, 60 µL of the diluted supernatant was incubated with 60 µL of RBD-HRP solution at 37 °C for 30 min. Following the incubation, 100 µL of each mixture was transferred to individual wells. The different RBD-HRP/MSC-supernatant ratios were as follows: ratio I (4:2): 50 µL of RBD-HRP, 25 µL of MSC supernatant, and 25 µL of dilution buffer; ratio II (4:3): 50 µL of RBD-HRP, 37.5 µL of MSC supernatant, and 12.5 µL of dilution buffer.

To quantify the binding of the RBD to hACE2, the kit uses the RBD conjugated with a peroxidase enzyme that catalyzes the degradation of the 3,3′,5,5′-tetramethylbenzidine (TMB) substrate, with the reaction intensity reflecting the RBD binding to ACE2 in the wells. The color intensity was inversely proportional to the RBD-neutralization activity of the BP2-containing supernatant added to the wells. The samples were then plated in triplicate on a 96-well plate included in the kit and incubated for 15 min at 37 °C. After washing, TMB solution was added, and the plates were incubated at room temperature (18–25 °C) for 25 min in the dark. A stop solution was then added to each well, and the absorbance was read immediately at 450 nm. Each run was validated according to predefined criteria provided by the kit [Arévalo-Romero et al. 2024]. RBD proteins from several SARS-CoV- 2 variants, including Wuhan, Mu, Omicron BA.1, and Omicron BA.2, were evaluated in these assays.

### Statistical analysis

The results were analyzed via GraphPad Prism v.10. The statistical significance of BP2-MSC culture supernatant neutralization activity compared with that of wtMSCs was assessed via one-way ANOVA, followed by Dunnett's multiple comparison test.

## Results and discussion

To date, there are no highly effective therapies or standardized protocols established for severe COVID- 19 treatment. Even though the pandemic has concluded, the search for effective prophylactic and therapeutic strategies continues to be critically important (Ceja-Gálvez et al. 2023). The SARS-CoV- 2 virus is endemic worldwide (Phillips 2021), and immunocompromised patients are at greater risk of infection and coinfections, increasing the likelihood of severe disease progression and poor outcomes (Li et al. 2024). Moreover, prolonged SARS-CoV- 2 infections in immunosuppressed patients can lead to the emergence of highly mutated variants (Raglow et al. 2024; Bansal et al. 2022; Weigang et al. 2021), posing significant challenges. These molecular alterations may confer an evolutionary advantage to the virus, allowing it to evade host immune defenses and complicating treatment strategies. Consequently, they could reduce the effectiveness of current vaccines (Greaney et al. 2021; Xue et al. 2024) as well as monoclonal antibodies (mAbs) developed against earlier SARS-CoV- 2 variants (Cao et al. 2022; Schoefbaenker et al. 2024; Choudhary et al. 2024; Planas et al. 2024).

To address this, new strategies have emerged, including in silico protein engineering to increase the affinity of soluble hACE2 for the SARS-CoV- 2 spike protein or its receptor-binding domain (RBD) (Linsky et al. 2020; Chan et al. 2020; Rakhmetullina et al. 2024). The aim of this approach is to improve hACE2 as a competitive inhibitor, blocking the interaction of the virus with membranous hACE2 and thereby reducing cellular entry and infection. However, new Omicron subvariants (Müller et al. 2024) continue to raise new alarms after exhibiting extensive immune evasion (Raisinghani et al. 2024; Zhao et al. 2024), which is associated with potential infection of lung cells (Zhang et al. 2024). MSCs, with their pulmonary homing signals, tissue repair ability and immunomodulation capacity, are used to combat lung injuries caused by severe COVID- 19 (Leng et al. 2020; Hashemian et al. 2021). Recently, MSCs have been used as vehicles to secrete SARS-CoV- 2-neutralizing mAbs, combining their immunomodulatory and tissue repair characteristics with the SARS-CoV- 2-neutralizing activity of mAbs to increase their therapeutic potential (Wang et al. 2023).

In a previous study, we demonstrated the neutralizing potential of three miniACE2 peptides (BP2, BP9, and BP11) against various SARS-CoV- 2 variants, including omicron subvariants (Arévalo-Romero et al. 2024). In this work, Wharton's jelly derived MSCs from three different donors (L100922000915, L100922000923 and L10092200996) at passage 3 were genetically engineered to secrete BP2, BP9 or BP11. Preliminary assays revealed that the culture supernatants of BP9-MSCs and BP11-MSCs, which were selected for their high puromycin resistance, exhibited no neutralization activity (Supplementary data 1). However, the culture supernatant of BP2-MSCs engineered using the pEF-BP2-PGK-P lentiviral vector (Fig. 1) demonstrated significant neutralizing activity. As reported by Arévalo-Romero, the BP2 peptide contains two glycosylation sites that are also present in the full-length wild-type ACE2 receptor. However, these glycosylation sites were mutated in the BP9 and BP11 peptides (Arévalo-Romero et al. 2024). The absence of glycosylation in BP9 and BP11 could explain their lack of secretion, as this post-translational modification plays a crucial role in the secretory pathway (Qu et al. 2006; Sagt et al. 2000).

**Fig. 3:**
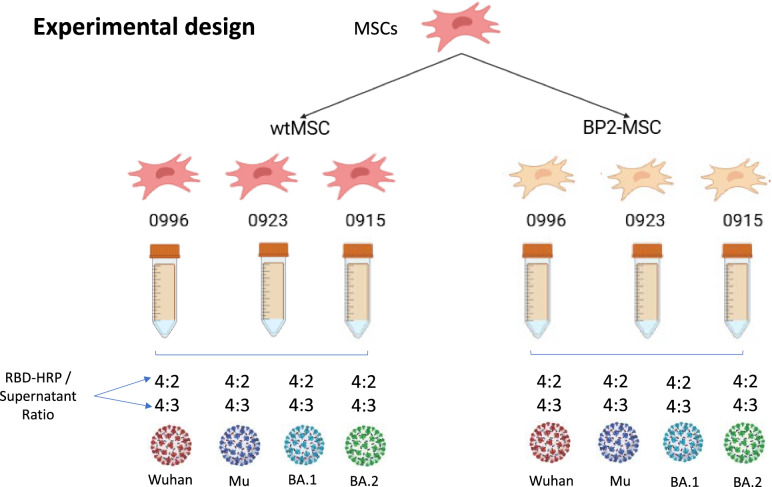
Schematic representation of the experimental design for generating MSC lines engineered to secrete the BP2 protein and evaluating the SARS-CoV- 2 neutralizing activity of BP2 in the MSC culture supernatant. The neutralization assay was performed via cPass™ SARS-CoV- 2 kits

This genetic engineering yielded three MSC strains that secreted the BP2 protein (BP2-MSC0915, BP2-MSC0923 and BP2-MSC0996). The wtMSCs and BP2-MSCs were thawed and cultured in T75 flasks with medium supplemented with FBS. The cells were allowed to grow to 60–90% confluence. At this point, the supernatant was removed, and the monolayer was washed once with serum/hPL-free medium to eliminate dead cells. Both wtMSCs and BP2-MSCs were subsequently characterized via flow cytometry to confirm the expression of specific MSC markers. Immunophenotyping was performed using Wharton’s jelly derived MSCs from three different donors, and the expression of CD73, CD90, HLA-I, CD105, CD34, CD45, and HLA-DR was assessed in accordance with the ISCT criteria. CD31 staining was used to exclude endothelial cell contamination, ensuring the identity and phenotypic integrity of the modified cell lines for downstream analyses (Dominici et al. 2006). Histograms were created to illustrate the overlapping expression of each marker in wild-type MSCs (blue) and BP2-transduced MSCs (red), confirming that the immunophenotype remained unchanged following transduction (Fig. 3). Then, the cells were cultured for an additional 4 days in serum/hPL-free medium. The supernatants were then collected for use in neutralization experiments (Fig. 2).

**Fig. 4:**
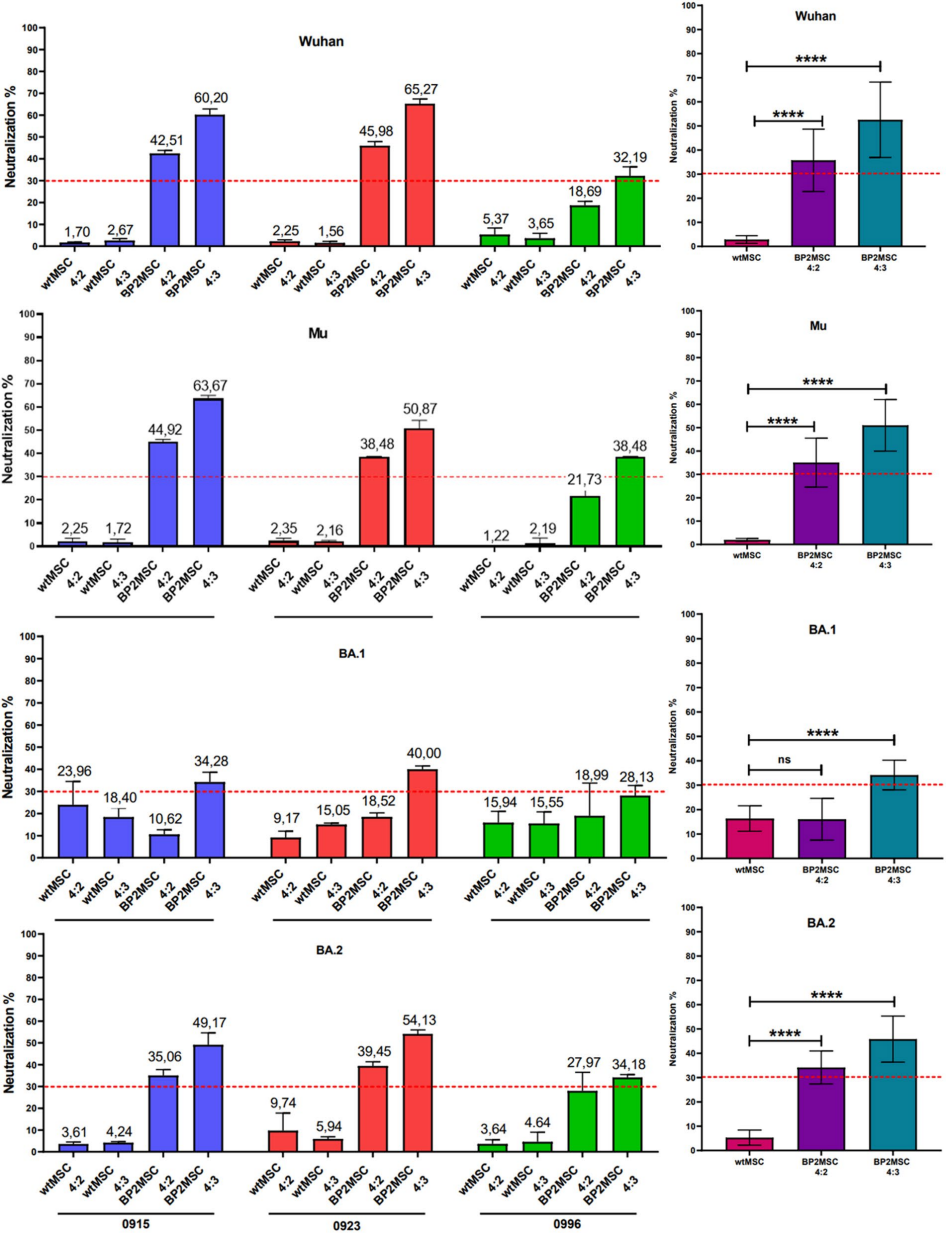
Neutralization performance of the supernatants with the Wuhan, Mu, BA.1 and BA.2 variants, showing the different donor and WT samples, with different RBD-HRP/supernatant ratios of 4:2 and 4:3. The dashed red line represents the neutralization threshold for the assay, which was set at 30%, as specified by the manufacturer

In these experiments, we observed the highest neutralization activity with the supernatants from BP2-MSCs at a 4:3 ratio derived from BP2-MSC0915 and BP2-MSC0923 against the Wuhan (60,20% and 65,27%, respectively), Mu (63,67% and 50,87%, respectively), and BA.2 (49,17% and 54,13%, respectively) SARS-CoV- 2 variants. As expected, the supernatants from wtMSCs, which were used as controls, did not exceed the established neutralization threshold in the SARS-CoV- 2 ELISA (Table 1, Fig. 4).

**Table 1:** Average neutralization activity of BP2-MSC culture supernatants compared with that of wtMSC culture supernatants

SARS-CoV- 2 variant →		Neutralization average							
		Wuhan		Mu		BA.1		BA.2	
		RBD/Supernatant ratio		RBD/Supernatant ratio		RBD/Supernatant ratio		RBD/Supernatant ratio	
Donor ↓		4:2	4:3	4:2	4:3	4:2	4:3	4:2	4:3
wtMSC0915		1.70	2.67	2.25	1.72	23.96	18.40	3.61	4.24
BP2-MSC0915		45.51	60.20	44.92	63.67	10.62	34.28	35.06	49.17
wtMSC0923		2.25	1.56	2.35	2.16	9.17	15.05	9.74	5.94
BP2-MSC0923		45.98	65.27	38.48	50.87	18.52	40.00	39.45	54.13
wtMSC0996		5.37	3.65	1.22	2.19	15.94	15.55	3.64	4.64
BP2-MSC0996		18.69	32.19	21.73	38.48	18.99	28.13	27.97	34.18

In contrast, the supernatant from BP2-MSC0996 exhibited significantly lower neutralization activity against all the tested SARS-CoV- 2 variants. This result could be attributed to impaired cell growth post-transduction, as these donor cells showed reduced confluence and slower proliferation than the other cell strains did (Fig. 4; supplementary data 1). Consequently, the supernatants of the BP2-MSC0996 cultures presented lower neutralizing activity. In any case, although the reduced levels may be due to poor cell growth, a low genomic copy number of the BP2 gene or a combination of both factors, neutralization levels above the minimum of 30% were achieved in the assays with the Wuhan, Mu and BA.2 variants at a 4:3 ratio (Table 1, Fig. 4). Moreover, the supernatants from wtMSCs derived from donors L100922000915 and L100922000923 presented higher neutralization values against the BA.1 variant than against the other variants. However, these values remained below the 30% test threshold, possibly due to nonspecific binding in the supernatant or experimental variability (Table 1, Fig. 4).

Finally, the neutralization effect of supernatants from BP2-MSCs was highly significantly different from that of supernatants from wtMSCs at a 4:3 RBD-HRP/supernatant ratio for all tested SARS-CoV- 2 variants. However, at a 4:2 ratio, no statistical significance was observed for the BA.1 Omicron variant (Table 1, Fig. 4).

## Conclusions

Our experiments explored the potential of recombinant proteins as broad-spectrum treatments or protective barriers against SARS-CoV- 2 and other ACE2-domain viruses. The BP2 protein secreted by BP2-MSCs was associated with an approximately 50% neutralization efficacy against the four tested variants. Using MSCs as a platform to secrete these proteins offers several advantages. In addition to their ability to secrete proteins, MSCs, owing to their immunomodulatory and regenerative properties, can help reduce damage to lung tissue. This approach shows promise as a therapeutic tool to support patient recovery and mitigate the effects of severe infection, especially in immunocompromised populations.

MSCs are increasingly recognized and approved as cellular therapies worldwide (Fernández-Garza et al. 2023; Blanc et al. 2025), their application as a treatment for SARS-CoV- 2 remains under investigation. Current perspectives indicate that MSC-based therapies could soon emerge as a novel approach to counteract the effects of viral infections, offering a promising avenue for cellular therapies targeting virus-induced damage.

## Supplementary Information

Additional file 1.

## Data Availability

No datasets were generated or analysed during the current study.

## Abbreviations

MSCs: Mesenchymal stromal cells
wtMSCs: Wild-type mesenchymal stromal cells
ACE2: Angiotensin-converting enzyme 2
mAbs: Monoclonal antibodies
PBS: Phosphate-buffered saline
P/S: Penicillin‒streptomycin
ISCT: International Society for Cell Therapy
hEF- 1α: Human eukaryotic translation elongation factor 1 alpha
APOA2: Apolipoprotein A2
hPGK: Human phosphoglycerate kinase
LTV: Lentiviral transfer vector
hPL: Human platelet lysate
TMB: 3,3′,5,5′-Tetramethylbenzidine
